# Supplementary material for: Do the Causes of Spontaneous Preterm Delivery Affect Placental Inflammatory Pathology and Neonatal Outcomes?
Source: Diagnostics (Basel). 2022 Sep 1;12(9):2126. doi: 10.3390/diagnostics12092126 (PMC9498177; doi:10.3390/diagnostics12092126)
Supplement: Supplementary file 1 [file diagnostics-12-02126-s001.zip › diagnostics-1836880-supplementary.pdf]

Supplementary Table S1. Comparison of severe histologic chorioamnionitis (HCA) or funisitis among women who delivered due to preterm labor (PTL), preterm premature rupture of membranes (PPROM) or incompetent internal os of cervix (IIOC).

	PTL vs. PPROM		PTL vs. IIOC		PPROM vs. IIOC	
	aOR* [95% CI]	p-value	aOR* [95% CI]	p-value	aOR* [95% CI]	p-value
Severe HCA	2.594 [1.660-4.054]	<0.001	1.368 [0.696-2.692]	0.364	0.554 [0.281-1.090]	0.554
Severe Funisitis	2.883 [1.824-4.559]	<0.001	1.587 [0.787-3.201]	0.197	0.601 [0.306-1.180]	0.139

* Adjustment for gestational age at delivery and admission to delivery interval
